# Preparation of a Microspherical Silver-Reduced Graphene Oxide-Bismuth Vanadate Composite and Evaluation of Its Photocatalytic Activity

**DOI:** 10.3390/ma9030160

**Published:** 2016-03-04

**Authors:** Mao Du, Shimin Xiong, Tianhui Wu, Deqiang Zhao, Qian Zhang, Zihong Fan, Yao Zeng, Fangying Ji, Qiang He, Xuan Xu

**Affiliations:** 1 Key Laboratory of Three Gorges Reservoir Region’s Eco-Environment, Ministry of Education, Chongqing University, Chongqing 400045, China; dumaozb6061@163.com (M.D.); 18512341504@163.com (S.X.); tianhuiwu109@163.com (T.W.); a2006silent@foxmail.com (D.Z.); zhangqianswu2005@163.com (Q.Z.); jfy@cqu.edu.cn (F.J.); 15996947229@163.com (Q.H.); 2 Chongqing Key Laboratory of Environmental Materials and Remediation Technology, Chongqing University of Arts and Sciences, Chongqing 400045, China; 3 College of Environmental and Resources, Chongqing Technology and Business University, Chongqing 400067, China; zhfan616@gmail.com; 4 Environmental monitoring station of Dadukou District, Chongqing 400084, China; xingxingrelei@126.com; 5 National Centre for International Research of Low-carbon and Green Buildings, Chongqing University, Chongqing 400045, China

**Keywords:** photocatalyst, Ag-reduced graphene oxide-BiVO_4_, characterization, degradation

## Abstract

A novel Ag-reduced graphene oxide (rGO)-bismuth vanadate (BiVO_4_) (AgGB) ternary composite was successfully synthesized via a one-step method. The prepared composite was characterized by X-ray diffraction (XRD), X-ray photoelectron spectroscopy (XPS), scanning electron microscopy (SEM), energy dispersive X-ray (EDX), Brunauer-Emmett-Teller (BET) surface area measurement, Raman scattering spectroscopy, and ultraviolet-visible diffuse-reflection spectroscopy (UV-vis DRS). The results showed that bulk monoclinic needle-like BiVO_4_ and Ag nanoparticles with a diameter of approximately 40 nm formed microspheres (diameter, 5–8 μm) with a uniform size distribution that could be loaded on rGO sheets to facilitate the transport of electrons photogenerated in BiVO_4_, thereby reducing the rate of recombination of photogenerated charge carriers in the coupled AgGB composite system. Ag nanoparticles were dispersed on the surface of the rGO sheets, which exhibited a localized surface plasmon resonance phenomenon and enhanced visible light absorption. The removal efficiency of rhodamine B dye by AgGB (80.2%) was much higher than that of pure BiVO_4_ (51.6%) and rGO-BiVO_4_ (58.3%) under visible light irradiation. Recycle experiments showed that the AgGB composite still presented significant photocatalytic activity after five successive cycles. Finally, we propose a possible pathway and mechanism for the photocatalytic degradation of rhodamine B dye using the composite photocatalyst under visible light irradiation.

## 1. Introduction

In recent years, bismuth vanadate (BiVO_4_) has received considerable attention as a promising photocatalyst with a narrow band gap compared with TiO_2_ [1]. Researchers have reported many merits of monoclinic BiVO_4_. First, monoclinic BiVO_4_, which has a relatively narrow band gap (2.4 eV), has a characteristic visible light absorption band in addition to the UV absorption band. Therefore, monoclinic BiVO_4_ is one of the most promising visible light-driven photocatalysts and has been widely studied for use in photodegradation of pollutants and in solar energy conversion [2,3]. In addition, it has effective masses of electrons and holes, which offers advantages for improving the separation efficiency of photogenerated charges [4]. Finally, it has the advantages of being low cost, environmentally friendly, and highly resistant to photocorrosion [5]. However, BiVO_4_ also has many disadvantages that have limited its widespread application for photocatalytic degradation of organic contaminants. For example, it has low photocatalytic activity as pure BiVO_4_, and most importantly, electron–hole recombination occurs with oxidation–reduction reactions, reducing its effectiveness for photocatalytic degradation of organic contaminants [6]. Researchers have made great efforts to solve this problem, such as incorporating reduced graphene oxide into the monoclinic BiVO_4_ [7,8].

Graphene oxide (GO) is used as a carbon additive in semiconductor photocatalysis, because it has several valuable characteristics [9]. First, GO-hybridized materials exhibit high electron mobility (200,000 cm^2^/V) and extended π-electron conjugation, and thus, GO is a good material for transporting electrons and stabilizing extraneous electrons. In addition, graphene has a high specific surface area (2630 m^2^/g) [10,11]. GO has been identified as a suitable candidate for creating the charge trapping layer for memory applications. Moreover, the band gap of GO can be tuned by simply varying the oxidation level. Fully oxidized GO can act as an electrical insulator, whereas partially oxidized GO can act as a semiconductor [9,12]. These properties of GO are good for decreasing the electron–hole recombination rate, which gives GO composites with BiVO_4_ better photocatalytic activity than pure BiVO_4_ [13,14]. To date, many studies have reported reduced graphene oxide (rGO)-BiVO_4_ composites and their enhanced photocatalytic activity [15]. Nevertheless, there is still a need to increase the photocatalytic activity of composites either by promoting light absorption or reducing the electron–hole recombination rate by incorporating other species into the binary composites [16].

It has been reported that the separation of electron–hole pairs can be improved by charge transfer between the semiconductor and metal. Hence, loading noble metals on semiconductor photocatalysts can effectively increase their photocatalytic performance [17,18]. Compared with other metals, Ag nanoparticles are a popular choice because they offer good ability to generate surface plasmons at the desired wavelength [19,20,21]. Moreover, Ag nanoparticles may exhibit a localized surface plasmon resonance (LSPR) phenomenon that enables them to have strong and broad absorption in the visible region of the solar spectrum. These factors, and especially the decrease in the recombination rate of the photogenerated charge carriers together with broad absorption of visible light, can induce a visible light-driven reaction for photocatalysis in BiVO_4_ [22]. Nevertheless, there have been no studies reporting the synthesis of a rGO and monoclinic BiVO_4_ composited embedded with Ag nanoparticles, and thus, the photocatalytic activity of such composites has not been reported.

We previously reported the single-step synthesis of BiVO_4_-reduced graphene nanocomposites that exhibited good visible light photocatalytic activity [23]. In the present study, we introduce a one-step method for the synthesis of Ag-rGO-BiVO_4_ (AgGB) composites. Our results demonstrate that bulk monoclinic needle-like BiVO_4_ and Ag nanoparticles with a diameter of approximately 40 nm formed microspheres (diameter, 5–8 μm) with a uniform size distribution that could be loaded on rGO sheets to facilitate the transport of electrons photogenerated in BiVO_4_, thereby reducing the rate of recombination of photogenerated charge carriers in the coupled AgGB composite system. In addition, photodegradation of rhodamine B (RhB) dye by AgGB was more efficient than by pure BiVO_4_ and rGO-BiVO_4_ under visible light irradiation. Finally, the AgGB composite still presented significant photocatalytic activity after five successive cycles, confirming the stability of its photocatalytic activity.

## 2. Materials and Methods

### 2.1. Experimental Materials

The following analytically pure chemicals were used: graphene oxide solution (≥99.85%, Shanghai HuaYi Company, Shanghai, China), bismuth nitrate (Bi(NO_3_)_3_·5H_2_O, Chengdu Area of the Industrial Development Zone Xinde Mulan), silver nitrate (AgNO_3_, 99.0%, Chongqing Chuandong Chemical Company, Chongqing, China), 25% ammonia solution (NH_3_**·**H_2_O, Chongqing Chuandong Chemical Company, Chongqing, China), sodium hydroxide powder (NaOH, Chongqing Chuandong Chemical Company, Chongqing, China), N,N-dimethylformamide (DMF, Chengdu Area of the Industrial Development Zone Xinde Mulan, Chengdu, China), ammonium metavanadate (NH_4_VO_3_, Chongqing Chuandong Chemical Company, Chongqing, China), nitric acid (HNO_3_, Chengdu Area of the Industrial Development Zone Xinde Mulan, Chengdu, China), rhodamine B (RhB) dye (Tianjin Guangfu Fine Chemical Research Institute, Tianjin, China), and ethylene glycol (Chongqing Chuandong Chemical Company, Chongqing, China).

### 2.2. Synthesis of AgGB Composite

In a typical experiment, 0.01 mol Bi(NO_3_)_3_·5H_2_O was dissolved in HNO_3_ solution, and 0.01 mol NH_4_VO_3_ was dissolved in NaOH solution. These two solutions were combined together and stirred vigorously for 30 min. The pH of the mixed solution was adjusted to 7 with NH_3_ solution. Separately, 8.2 mL of GO solution (2.0 mg/mL) was sonicated in an ultrasonic bath for about 1 h. After 1 h, the GO solution was added to the BiVO_4_ mixed solution. At the same time, 0.6478 g AgNO_3_ powder dissolved in 10 mL EG was added to the BiVO_4_ mixed solution, and the resulting solution was sonicated for another 1 h. Afterward, a homogeneous suspension formed, and the obtained sample was transferred to a 50-mL Teflon-sealed autoclave and maintained at 474.15 K for 18 h to achieve crystallization of the AgGB composite. The precipitate was centrifuged and washed with deionized water eight times and dried in the vacuum freeze drier at −60 °C for 24 h. Thus, all the composites contained the same amount of BiVO_4_ and Ag. The synthesized ternary AgGB composite was named AgGB-x, where x is the mass percentage of rGO. For comparison, the same procedure was followed to synthesize BiVO_4_-rGO, a binary composite of BiVO_4_ and rGO that is denoted as Bi-rGO.

### 2.3. Characterization

Powder X-ray diffraction (XRD) spectra were acquired with a Rigaku D/Max-rB diffractometer with Cu Kα radiation. The 2θ scanning angle ranged from 15° to 70°. Scanning electron microscopy (SEM) images were acquired with a JSM-7800F JEOL emission scanning electron microscope. Energy dispersive X-ray (EDX) images were acquired with an EDX-100A-4. Raman spectra were recorded on an HR Evolution instrument with an Ar+ laser source of 488 nm. The Brunauer-Emmett-Teller (BET) specific surface areas and porosity of the samples were evaluated on the basis of nitrogen adsorption isotherms measured at 400 °C using a gas adsorption apparatus (Gemini VII 2390, Micromeritics Instrument Corp, Norcross, GA, USA). The samples were degassed at 400 °C before nitrogen adsorption measurements. The BET surface area was determined using adsorption data in the relative pressure (p/p_0_) range of 0.05–1. X-ray photoelectron spectroscopy (XPS) characterization was performed (K-Alpha, UA, Thermo Fischer Scientific, Waltham, MA, USA) with an Al Ka X-ray source. All binding energy values were corrected by calibration to the C 1s peak at 284.6 eV. UV-visible diffuse-reflectance spectroscopy (UV-vis DRS) was performed with a Hitachi U-3010 UV-vis spectrometer.

### 2.4. Evaluation of Photocatalytic Activity

The photocatalytic activity of the AgGB composite was assessed by evaluating the photodegradation of a RhB solution under illumination of visible light at room temperature. A 500 W Xe lamp (λ ≥ 400 nm) was used as the visible light irradiation source. For the experiments, 0.20 g catalyst was first added to 200 mL of a 5 mg/L RhB aqueous solution. Before irradiation, the mixture was magnetically stirred for 30 min in the dark to obtain good dispersion and reach adsorption–desorption equilibrium between dye and catalyst. The experimental solution was placed in a 250-mL beaker 350 mm away from the light source. After 1-h sessions of irradiation, 8-mL aliquots were withdrawn and centrifuged at 10,000 r/min to remove essentially all catalyst. The concentration of the remaining dye was spectrophotometrically monitored by measuring the absorbance of the solutions at 552 nm. For comparison, the photocatalytic experiments were carried out with Bi-rGO, Bi-Ag, or pure BiVO_4_ as the catalyst as well as in the absence of any catalyst under the same conditions.

## 3. Results and Discussion

### 3.1. Pattern Analysis: XRD

**Figure 1:**
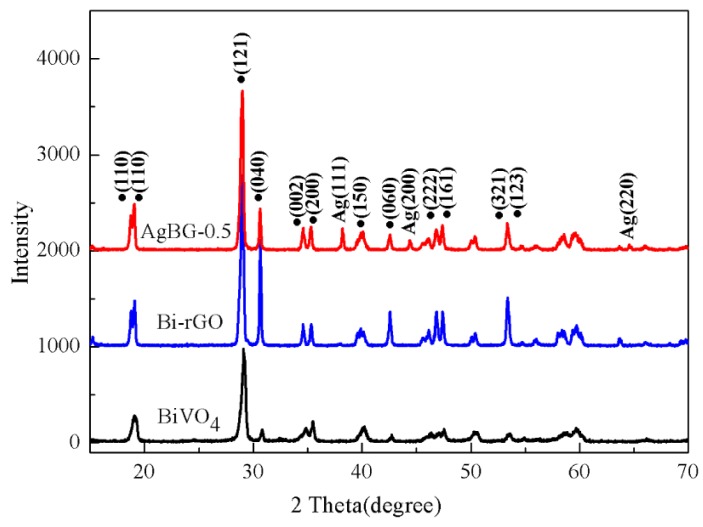
XRD patterns of pure BiVO_4_ and Bi-rGO and AgGB-0.5 composites.

**Figure 2:**
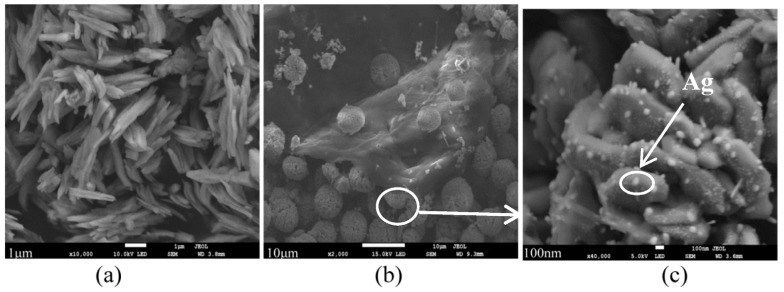
SEM images of: (**a**) pure BiVO_4_; (**b**) AgGB-0.5; and (**c**) AgGB-0.5 at higher magnification.

The phase structures of the composites were characterized by X-ray diffraction (XRD) measurements, and the XRD patterns of the pure BiVO_4_, Bi-rGO, and AgGB-0.5 are shown in Figure 1. Almost all the diffraction peaks of the BiVO_4_ could be assigned to monoclinic BiVO_4_ (JCPDS 14-0688), which is the most active photocatalyst under visible light irradiation [24]. Compared with the curves of BiVO_4_ and Bi-rGO, the diffractogram of the AgGB showed some new peaks. The peaks at 38.1°, 44.3°, and 64.51° were assigned to the (111), (200), and (220) planes of face centered cubic (FCC) Ag (JCPDS card no. 65-2871), respectively [25]. The XRD analysis revealed that the phase of BiVO_4_ did not change after the addition of the AgNO_3_ and GO solution. This result proves that AgNO_3_ successfully reduced and was transformed by Ag nanoparticles with an average dimension of 40-nm (see Figure 2c) under the solvothermal conditions.

### 3.2. Morphology and Composition Analysis: SEM, EDX, and Raman Scattering Spectra

The sizes and morphologies of the prepared samples were examined by scanning electron microscopy (SEM). Figure 2 shows SEM images of different samples. As shown in Figure 2a, pure BiVO_4_ particles had uniform size and shape distributions and formed a series of needle-like structures. However, the image in the Figure 2a also shows some BiVO_4_ aggregates. Figure 2b shows that some microspheres were loaded on the rGO sheets and some Ag nanoparticles were dispersed on the surface of the rGO sheets. Single microspheres had diameters ranging from 5–8 μm. Figure 2c shows a magnified image of a single microsphere, and it can be observed clearly that Ag nanoparticles with a diameter of approximately 40 nm were uniformly dispersed on the surface of BiVO_4_. This structure creates a good interface between BiVO_4_, Ag nanoparticles, and rGO to facilitate efficient charge transport within the composite, which leads to efficient separation of photogenerated carriers in the coupled rGO composites [3]. Some Ag nanoparticles were dispersed on the surface of the rGO sheets, which exhibited a LSPR phenomenon and enhanced absorption of the visible light [16].

**Figure 3:**
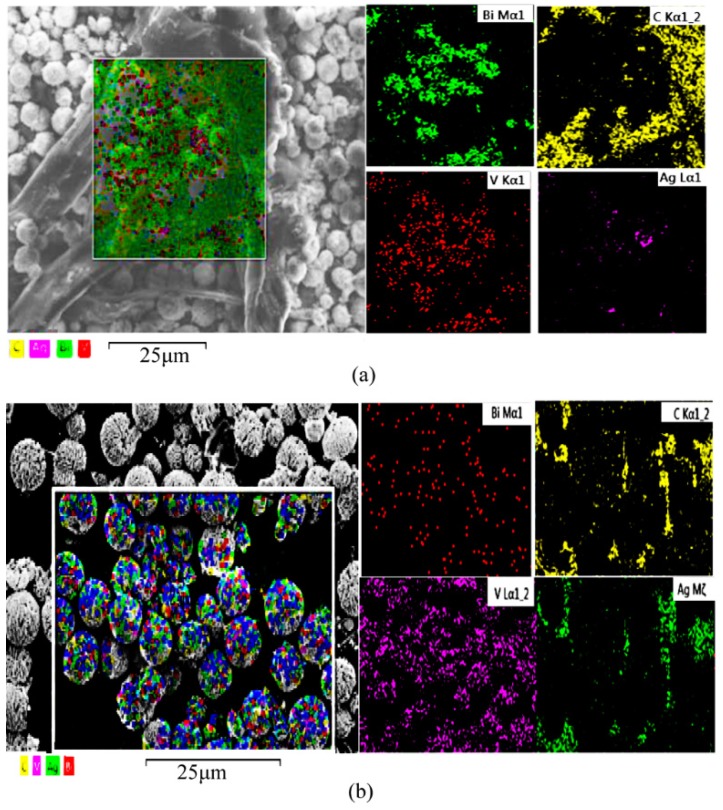

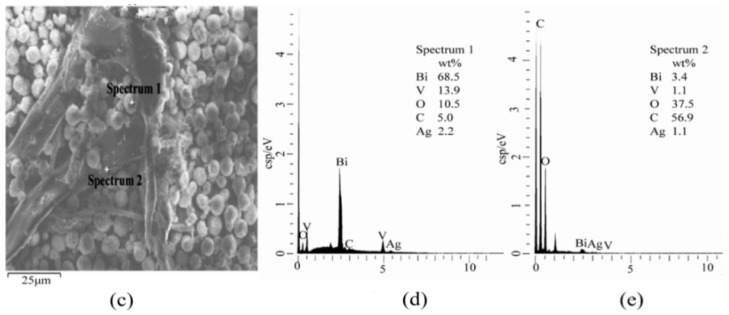
EDX spectrum of the AgGB-0.5 composite and the corresponding EDX elemental mapping results: (**a**) AgGB-0.5 composite; (**b**) BiVO_4_ and Ag microspheres; (**c**) AgGB-0.5 composite; and (**d**,**e**) the corresponding EDX elemental mapping results of AgGB-0.5 composite.

The EDX spectrum of the AgGB-0.5 composite was measured to assess its chemical composition (Figure 3) and showed that the composite was composed mainly of Ag, Bi, C, O, and V elements. Comparing Figure 3a,b, the ratio of C was increased, which combined with the XPS analysis enabled us to draw the following conclusion: the surfaces of BiVO4 and Ag are covered with a layer of rGO film. The corresponding EDX spectra (Figure 3d,e) exhibited the characteristic peaks of elemental C, Bi, V and Ag. A comparison of the spectra indicated that the uniformly distributed microspheres mainly consisted of BiVO4 and Ag nanoparticles. Together, the SEM and EDX results suggest that the BiVO4 and Ag nanoparticle composites were successfully coated on the surface of rGO film by this method. This observation is consistent with the XRD results.

**Figure 4:**
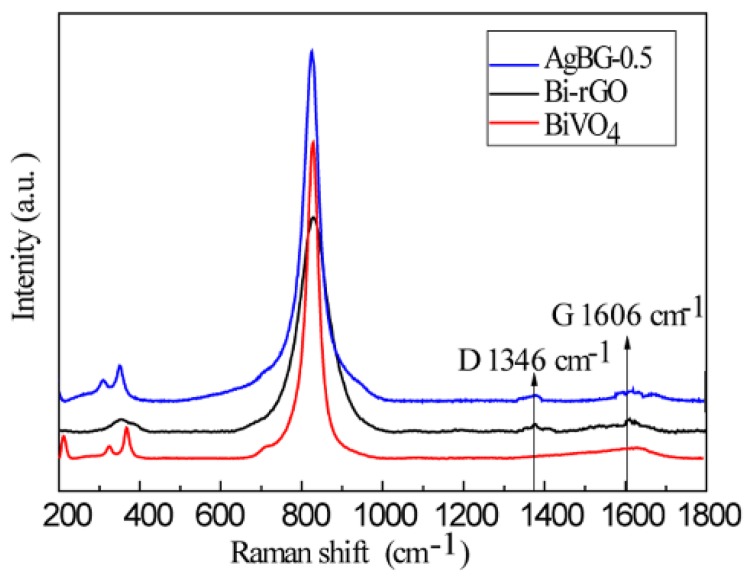
Raman spectra of the pure BiVO_4_ and Bi-rGO and AgGB-0.5 composites.

The structures of the as-prepared pure BiVO_4_ and Bi-rGO and AgGB-0.5 composites were further studied by Raman spectroscopy. As shown in Figure 4, the Raman spectrum of rGO displayed two prominent peaks at 1346 and 1606 cm^−1^, corresponding to the well-documented D and G bands, respectively [26]. The peaks at 820, 367, 324, and 210 cm^−1^ corresponded to the typical vibrations of monoclinic BiVO_4_. The peaks at 820 cm^−1^ were assigned to the typical symmetric and antisymmetric stretching modes of V-O bonds, whereas those at 367 and 324 cm^−1^ were attributed to the typical symmetric and antisymmetric bending modes of the vanadate anion [27]. The Raman spectrum of the AgGB-0.5 composite further confirmed the formation of rGO sheets.

### 3.3. Chemical States by XPS

**Figure 5:**
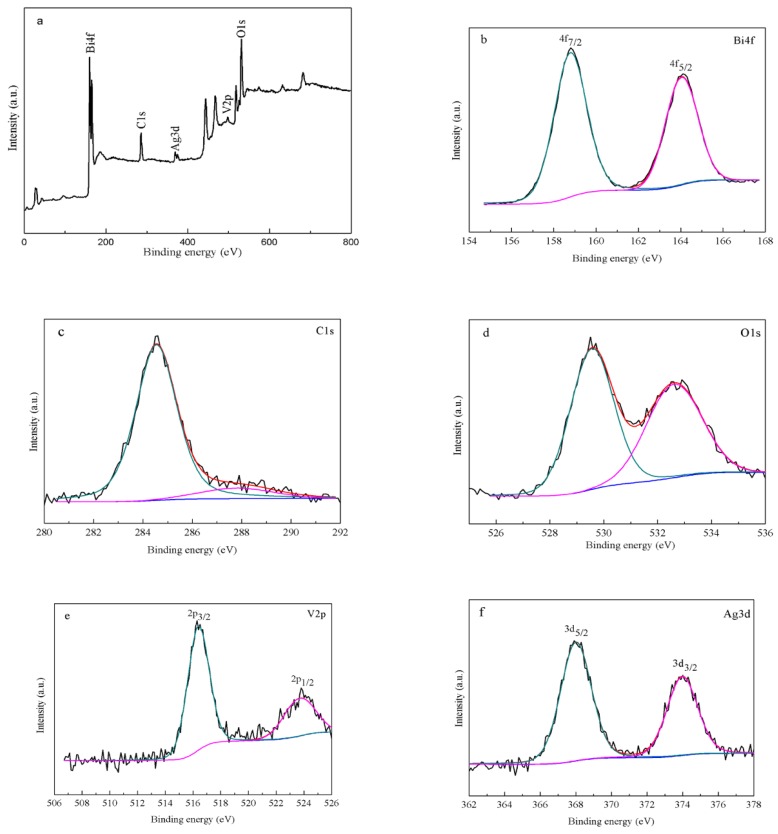
XPS of the as-obtained AgGB-0.5: (**a**) survey XPS spectrum; (**b**) Bi 4f spectrum; (**c**) C1s spectrum; (**d**) O1s spectrum; (**e**) V2p spectrum; and (**f**) Ag 3d spectrum.

The chemical and bonding environments of the successfully loaded AgGB-0.5 composite were evaluated by XPS (Figure 5). Figure 5a shows the fully scanned spectrum of the AgGB-0.5 composite in the range of 0–800 eV, and the survey spectrum showed that the composite contained Ag, Bi, O, V, and C. Figure 5b,e revealed that the binding energies were 158.8 and 164.1 eV for Bi 4f_7/2_ and Bi 4f_5/2_, respectively, which closely correspond to the Bi^3+^ peak in the monoclinic BiVO_4_ [28]. The C1s XPS spectrum showed two characteristic peaks, corresponding to oxygenated ring C bonds (284.6 eV for C-C, C=C and C-H, and 288.1 eV for the C=O bond). These results indicate that rGO surface contained abundant oxygen-containing functional groups. However, in the C 1s XPS spectra of AgGB-0.5 (Figure 5c), the relative intensities of the three components associated with C-O/C=O bonds decreased significantly, suggesting that some of the oxygen functional groups were reduced during the chemical reduction process [29,30]. The peaks at binding energies of 524.1 (V2p_1/2_) and 516.3 eV (V2p_3/2_) were the split signal of V2p (Figure 5e). The V2p peak is assigned to V^5+^ [31]. Figure 5f shows that the Ag3d peaks were centered at about 368.0 eV and 373.8 eV, and these were ascribed to Ag^0^ [32]. These results demonstrate that AgGB-0.5 composite was composed of BiVO_4_, rGO, and Ag nanoparticles, and the Ag nanoparticles were embedded in the rGO layer through strong interactions, which is supported by the LSPR of the Ag nanoparticles.

### 3.4. Optical Properties: UV-vis DRS

**Figure 6:**
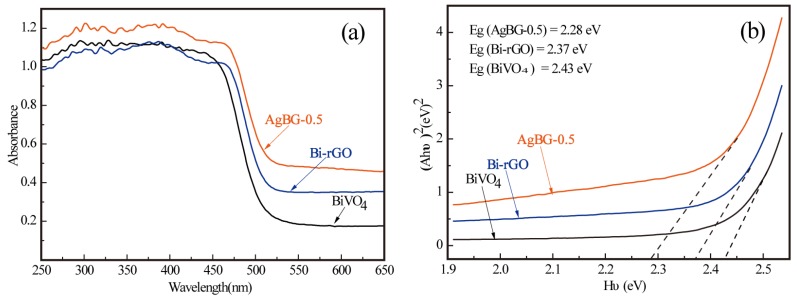
(**a**) UV-vis DRS spectra; and (**b**) the relationship between (Ahυ)^2^ and the photon energy (hυ) of the as-synthesized pure BiVO_4_, Bi-rGO, and AgGB-0.5.

We investigated the band edge position of the synthesized materials through UV-visible absorption spectroscopic analysis. Figure 6 shows representative spectra of pure BiVO_4_, the Bi-rGO composite, and the AgGB-0.5 composite. Notably, the AgGB-0.5 composite showed significant enhancement of light absorption at a wavelength of 400–800 nm, which could be mainly attributed to absorption of visible light by the rGO itself. The curve for the AgGB-0.5 composite showed that the high visible light absorption efficiency of the AgGB-0.5 composite was based on the synergistic effect of rGO and Ag nanoparticles. The absorption curve for the AgGB-0.5 composite was obviously enhanced in the visible light region, which could be attributed to the surface plasmon band of Ag nanoparticles. Surface plasmon absorption in the metal nanoparticles arises from the collective oscillations of the free conduction band electrons that are enhanced by the incident electromagnetic radiation [16]. This is sensitive to particle size, shape, and size distribution as well as the surrounding medium. The effect of surface plasmon resonance due to Ag nanoparticles partially contributed to the enhanced photocatalytic activity of the composite [33].

Moreover, the energy band structures of a semiconductor continue to be important in determining its photocatalytic activity. The relationship of absorbance and incident photon energy hυ can be described by Equation (1)

Ahυ = C(hυ − E_g_)^1/2^ (1)

where A, Eg, h, and υ represent the absorption coefficient, the band gap energy, Planck constant, and incident light frequency, respectively, and C denotes a constant [34,35]. The band-gap energy (Eg) of the obtained photocatalysts can therefore be estimated from a plot depicting (Ahυ)^2^ *versus* hυ. The estimated band-gap energies of pure BiVO_4_, Bi-rGO and AgGB-0.5 were measured to be 2.43 eV, 2.37 eV, and 2.28 eV, respectively. Notably, the absorption edge for the AgGB composite was red-shifted compared to those of pure BiVO_4_ and Bi-rGO, given that the absorption edges were measured to be at 510.29 nm for pure BiVO_4_, 523.21 nm for Bi-rGO, and 543.86 nm for AgGB-0.5.

### 3.5. Photocatalytic Activity for Degradation of RhB

**Figure 7:**
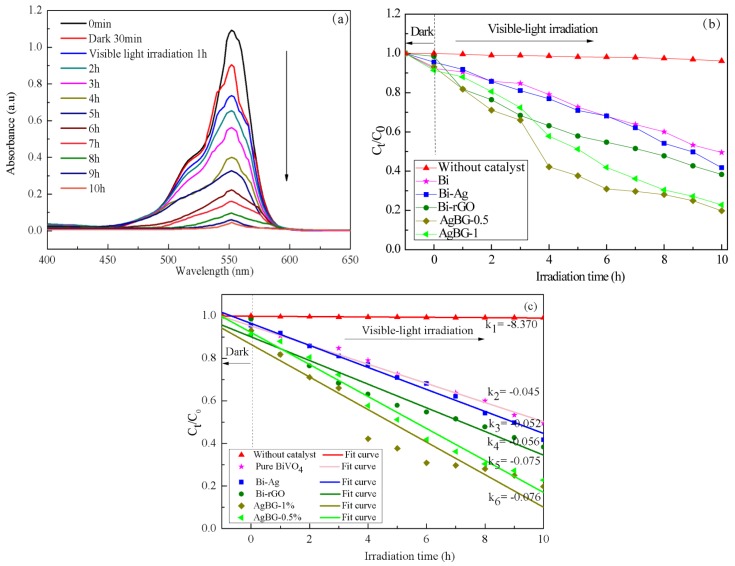
(**a**) Photocatalytic performance of AgGB composite for the degradation of RhB as measured by UV-vis DRS; (**b**) Degradation of RhB over different catalysts under visible light irradiation; (**c**) Photo-catalytic reaction with linear fitting modes and the reaction rate constant k.

Under visible light illumination, the photocatalytic activity of pure BiVO_4_, Bi-rGO, Bi-Ag, and AgGB was studied according to the degradation of RhB in solution (Figure 7). As a comparison, the photolysis of RhB was evaluated under the same conditions but without catalyst. It was found that only about 5% of RhB was decomposed after visible light irradiation for 10 h. Figure 7 also shows that the concentration of RhB gradually decreased as a result of visible light irradiation with catalysts. After visible light irradiation for 10 h, 51.6%, 58.3%, 62.7%, 80.2%, and 77.2% RhB was photocatalytically degraded with pure BiVO_4_, Bi-rGO, BiVO_4_-Ag, AgGB-0.5, and AgGB-1, respectively. It was clear that the photodegradation rates of RhB with Bi-rGO and AgGB were higher than those with pure BiVO_4_, as the AgGB with 0.5% rGO exhibited the best photocatalytic efficiency, which was because its larger BET surface area and pore volume (Table 1) are beneficial to contact of the AgGB composite with organic contaminants, which can enhance the photocatalytic performance after loading of the rGO sheets and Ag nanoparticles [35,36]. In addition, the rGO and Ag nanoparticles facilitated the transport of electrons photogenerated in the BiVO_4_, and therefore led to efficient separation of photogenerated carriers in the coupled Bi-rGO system [20,21]. Moreover, the photocatalytic degradation of RhB depended on the adsorption ability of BiVO_4_, and the Ag nanoparticles exhibited a LSPR phenomenon, which enabled them to have strong and broad absorption in the visible region of the solar spectrum. This increased the absorption of visible light on BiVO_4_, and the end result was an increase in the decomposition of RhB.

### 3.6. Postulated Mechanism of RhB Degradation over Photocatalyst

Under visible excitation, an electron of BiVO_4_ can be promoted from the valence band to the conduction band, leaving behind a hole in the valence band. With the addition of rGO to BiVO_4_, the generated electron on the surface of BiVO_4_ can be trapped by the rGO sheet. With the inclusion of Ag nanoparticles, photogenerated electrons can also be transported promptly by Ag nanoparticles, because the Fermi level of BiVO_4_ is higher than that of Ag [27]. Thus, the rate of electron–hole recombination is decreased. Therefore, both the added rGO and embedded Ag nanoparticles could enhance the separation of the photoinduced electron–hole pairs from BiVO_4_.

**Figure 8:**
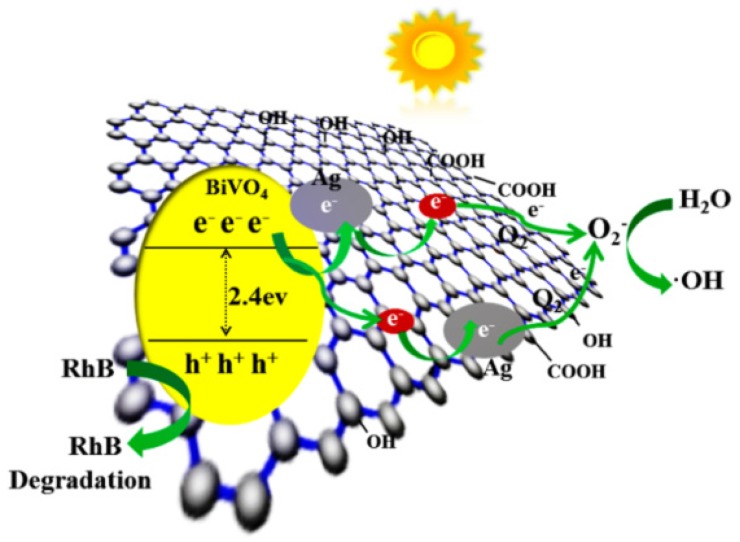
Photocatalytic reaction mechanism for AgGB composite.

According to the mechanism of degradation (as shown in Figure 8), upon visible light excitation, the BiVO_4_ surface generates electron–hole pairs, and this is followed by rapid transfer of photogenerated electrons to rGO sheets via a percolation mechanism. Then, the photogenerated electrons are transported on the surface of the Ag nanoparticles. Next, the negatively charged Ag nanoparticles react with dissolved oxygen molecules (O_2_) to yield superoxide radical anions (O_2_^·−^). The holes can react with OH^−^ to form OH, and finally, through a series of reactions with H^+^, the activated O_2_^·−^ further forms OH^−^ radicals, which are strong oxidizing agents for the decomposition of organic dyes [37,38]. The entire sequence is summarized as follows:

BiVO_4_ + hυ → BiVO_4_(h+e) (2)

BiVO_4_(e) + Ag → BiVO_4_ + Ag(e) (3)

Ag(e) + rGO → rGO(e) + Ag(4)

rGO(e) + O_2_ → O_2_^·—^ + rGO (5)

BiVO_4_(e) + rGO → BiVO_4_ + rGO(e) (6)

rGO(e) + Ag → rGO + Ag(e)(7)

Ag(e) + O_2_ → O_2_^·—^ + Ag (8)

BiVO_4_(h) + OH^—^ → BiVO_4_ + ·OH (9)

BiVO_4_(h) + ·OH+ O_2_^·—^+ RhB → degradation of RhB (10)

From Figure 8, it is clear that the photocatalytic efficiency will not increase with the addition of more rGO in the AgGB composite, possibly because too much rGO can lead to the formation of recombination centers for electrons and holes. This parallel recombination pathway reduces the probability that photoexcited charges participate in the photocatalytic reaction. As a result, a high rGO content reduces the photocatalytic activity.

**Figure 9:**
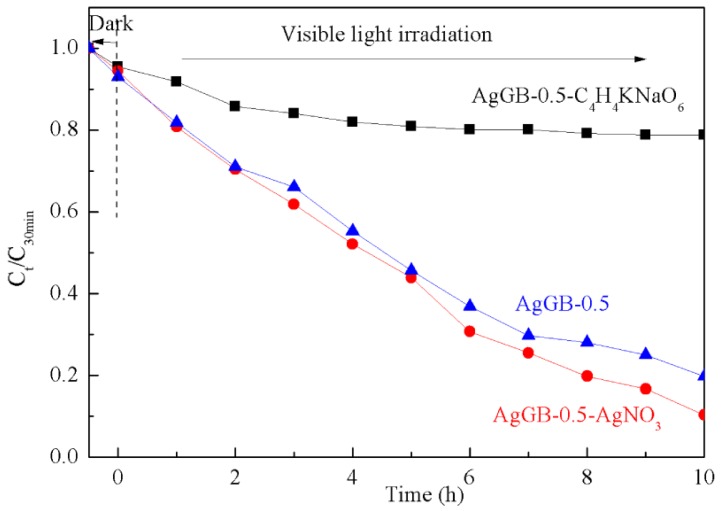
Photocatalytic degradation of RhB in AgGB-0.5 after addition of an electron-trapping agent or a hole-trapping agent.

To study the contribution of electrons and holes to the degradation reaction, silver nitrate (AgNO_3_, 0.1 mmol) and potassium sodium tartrate (C_4_H_4_O_6_KNa·4H_2_O, 0.1 mmol) were added to the AgGB-0.5 photocatalytic reaction system as an electron-trapping agent and hole-trapping agent, respectively. This approach allowed us to observe the degradation of RhB in the presence of either only electrons or only holes. As shown in Figure 9, adding silver nitrate clearly increased the degradation rate of RhB. In contrast, adding potassium sodium tartrate reduced the photocatalytic effect. These results suggest that the holes play the main role in the degradation of RhB in this system.

### 3.7. Recycling and Stability of AgGB Composite

**Figure 10:**
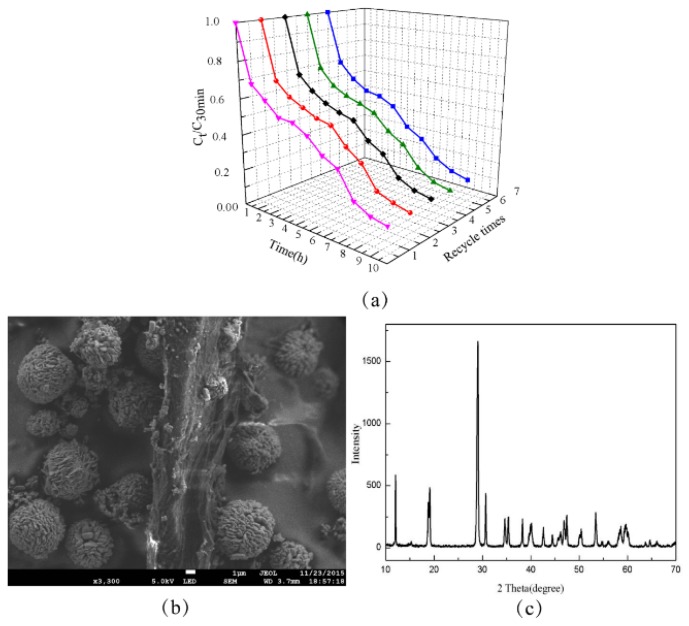
(**a**) Cycling runs of photocatalytic degradation of RhB over AgGB-0.5 photocatalyst; (**b**) SEM image of AgGB after five rounds of cycling; and (**c**) XRD patterns of AgGB-0.5 composite after five rounds of cycling.

As a catalyst material, the stability of AgGB composite is very important for its practical application. The stability of the AgGB composite was investigated through cyclic degradation of RhB under visible light irradiation, as shown in Figure 10a. The results indicate that the percentage of photocatalytic degradation was nearly constant over five cycles. In the repeated experiments, the AgGB sample was easily recycled by simple filtration without any additional treatment. As shown in Figure 10b, the composite does not exhibit any significant loss of activity except that the rGO sheets begin to aggregate after five successive cycles. Moreover, Figure 10c shows that the phase structures of the composite do not change, which confirms that the components of the AgGB are not photo decomposed and the structure is stable during the photocatalytic process. Thus, the stability of the AgGB composite is very good.

## 4. Conclusions

In conclusion, an AgGB composite was successfully prepared via a one-step method, and the composite was well characterized by a variety of instrumental techniques. Together, the results demonstrate that the AgGB composite exhibited the highest photocatalytic efficiency for RhB degradation compared to pure BiVO_4_, BiVO_4_-Ag, and Bi-rGO under visible light irradiation. The as-synthesized AgGB composite contains more photocatalytic reaction sites, which is not only attributed to the improved charge separation efficiency of the photogenerated electron–hole pairs in BiVO_4_, but also to the surface plasmon resonance and electron transfer effects of the Ag nanoparticles. The synthesized AgGB composite can easily be recycled without a decrease in photocatalytic activity due to its one-dimensional nanostructure. It is expected that the highly photocatalytic AgGB composite can be applied in industry to eliminate organic pollutants from wastewater.

## Figures and Tables

**Table 1:** Characteristics obtained from nitrogen desorption isotherms.

Sample	Mean Pore Size (nm)	Pore Volume (cm^3^g^−1^)	Surface Area (m^2^g^−1^)
Bi	3.7889	0.001958	1.3211
Bi-rGO	3.1674	0.003353	1.9372
AgGB-0.5	11.1333	0.026298	3.8862
AgGB-1	10.2568	0.023189	3.1494

## Abbreviations

The following abbreviations are used in this manuscript:

GO: graphene oxide
rGO: reduced graphene oxide
BiVO_4_: bismuth vanadate
Bi-rGO: bismuth vanadate-reduced graphene oxide
AgGB: A novel Ag-graphene oxide-bismuth vanadate
LSPR: localized surface plasmon resonance
RhB: rhodamine B dye
XRD: X-ray diffraction
SEM: scanning electron microscopy
EDX: energy dispersive X-ray
BET: Brunauer-Emmett-Teller
XPS: X-ray photoelectron spectroscopy
UV-vis DRS: UV-visible diffuse-reflectance spectroscopy
FCC: face centered cubic
